# An intelligent control algorithm for gas precise drainage problem based on Model Predictive Control

**DOI:** 10.1371/journal.pone.0332836

**Published:** 2026-02-03

**Authors:** Liyun Han, Yanli Wang

**Affiliations:** 1 School of Economics and Management, China University of Mining and Technology, Xuzhou, China; 2 School of International Education, Xi’an Peihua University, Xi’an, China; Henan Polytechnic University, CHINA

## Abstract

Intelligent extraction of coal seam gas constitutes a crucial development direction for managing underground gas disasters. Building on an established mathematical model, this study develops an intelligent control model for gas extraction. In this model, controlled variables include gas extraction concentration, gas extraction flow rate, negative pressure, and extraction pump efficiency ratio, while control variables are defined as the valve opening of extraction boreholes and the power of extraction pumps. The ideal curve of the controlled quantity with time is obtained by using the recurrent neural network (SimpleRNN), and the controlled quantity is intelligently controlled by the model predictive control (MPC) algorithm so that the actual value of controlled quantity approaches the reference value at the corresponding time of its ideal curve. Taking the simulated gas extraction data as an example, an algorithm simulation experiment is performed. The experimental results show that the ideal reference curve of the controlled quantity obtained by the cyclic neural network has a good data fitting degree. The dynamic control of the controlled quantity by the model predictive control algorithm can overcome the interference of environmental and nonlinear factors and achieve a better control effect, which provides a certain reference for the intelligent control of gas drainage.

## 1. Introduction

Gas drainage serves as an effective measure to address potential hazards in deep coal mining operations [1]. Intelligent management and control of gas extraction system has become an inevitable trend of mine disaster prevention and control in the process of coal mine intelligence, and it is also a realistic demand for efficient and accurate gas extraction in mines [2]. However, current gas extraction control remains heavily reliant on manual experience, preventing real-time and accurate regulation. Consequently, both the effectiveness and safety of gas extraction systems still need improvement.

Scholars and experts worldwide have conducted extensive research to enhance the safety and efficiency of underground gas extraction. Zhao [3], Wang [4], Ren [5] and Solomon A. Wassie [6] analysed the influencing factors in the process of gas extraction. Zhou [7,8] and Xia [9] put forward the principle of intelligent gas extraction based on the safety principle and efficiency principle of gas extraction, and they realized parameter monitoring and extraction control on the platform of computer and mobile terminal. Additionally, Liu [10], Yang [11], Ma [12], Li [13] and others carried out research to address the potential safety hazards of gas drainage and improve the security of gas drainage.

To realize intelligent underground gas extraction, many researchers have explored multi-parameter prediction for gas extraction and negative pressure regulation. Wu [14] built an improved GASA-BP prediction model by introducing an adaptive learning rate into the original BP neural network algorithm. Yuan [15] studied the theory and method of constructing and updating online data mining models based on feature selection strategies. Dong [16] extracted early warning information related to the evolution of atypical sudden disasters from gas concentration data and established a mathematical model for early warning indicators. Pan [17] constructed a tightness wavelet neural network toolbox to improve the accuracy of gas concentration prediction. Tong [18] constructed a gas concentration trend surface analysis model by data mining the mine gas concentration collected by wireless sensor network. Jia [19] fully leveraged the time-series characteristics of gas concentration data and proposed a mine gas concentration prediction model based on Gated Recurrent Units (GRUs). Pan [20] constructed a BP neural network surface subsidence prediction model based on Adaboost, which provides a certain basis for the multi-parameter change of mine gas. Therefore, further research on intelligent control models for gas extraction is essential to enhance the safety and efficiency of gas extraction operations.

An efficient intelligent control model relies on high-precision prediction data for relevant parameters. However, as the prediction time scale expands, the discrepancy between the actual and predicted gas extraction effects gradually increases. This issue makes it difficult for the control result to meet the actual operation requirements of gas extraction systems, and model predictive control (MPC) can effectively solve this problem [21–23].

MPC calculates and predicts the running state of the system over a limited time period in the future at each sampling time point and takes the current system state as the initial state. Then, it obtains the optimal control strategy at the next time through online rolling optimization so that the system state at each time infinitely approaches the ideal state curve [24,25]. This study focuses on developing an intelligent control model for gas extraction based on MPC. Specifically, it proposes four control tasks and corresponding theoretical control strategies for gas extraction systems. Under the joint constraints of safety and efficiency, an optimized mathematical model for gas drainage is established. A simple recurrent neural network (simple RNN) is used to generate the ideal time-varying curves of key characteristic parameters (gas extraction concentration, flow rate, negative pressure, and efficiency ratio). The MPC model is then applied to intelligently control gas extraction, with correction feedback and rolling optimization enhancing the anti-interference capability of the control system. This enables dynamic and intelligent optimization of the valve opening of extraction boreholes and the power of extraction pumps, ensuring that the key characteristic parameters of the gas extraction system closely align with their ideal curves at all times. Ultimately, this research aims to improve the safety and efficiency of coal mine gas extraction.

## 2. Gas intelligent extraction principle and control strategy

The gas extraction system is mainly composed of extraction pumps, pipeline distribution, valve control, data monitoring mechanism and other ancillary equipment. Intelligent gas extraction involves dynamically adjusting and matching the relevant working condition parameters under the guarantee of safety and efficiency. The parameters of the gas extraction system, such as the valve opening and pump power, are intelligently adjusted and controlled. When the negative pressure and temperature are within the safe threshold, the measurement indices of the gas concentration, gas purity and pump efficiency ratio are dynamically optimal.

Due to the instability in the gas extraction process, the gas extraction system has four major control tasks: (a) Increase gas extraction concentration to meet the requirements of efficient gas extraction; (b) Improve the pure quantity of gas extraction to meet the requirements of efficient gas extraction; (c) Maintain the extraction pump efficiency ratio within an ideal range to ensure economic extraction; (d) Keep gas drainage under reasonable negative pressure conditions to prevent gas-related safety accidents.

The four tasks of the gas extraction system affect each other and are an organic combination. The gas extraction concentration and gas extraction purity are affected by the valve opening and extraction pump power. The efficiency ratio of the extraction pump is closely related to the extraction pump power and gas extraction effect. A reasonable negative pressure condition is a prerequisite that must be met under the principle of safety. Therefore, this paper analyses relevant parameters and proposes an intelligent control model for gas extraction based on model predictive control [26–28].

### 2.1. Gas extraction system

**Fig 1:**
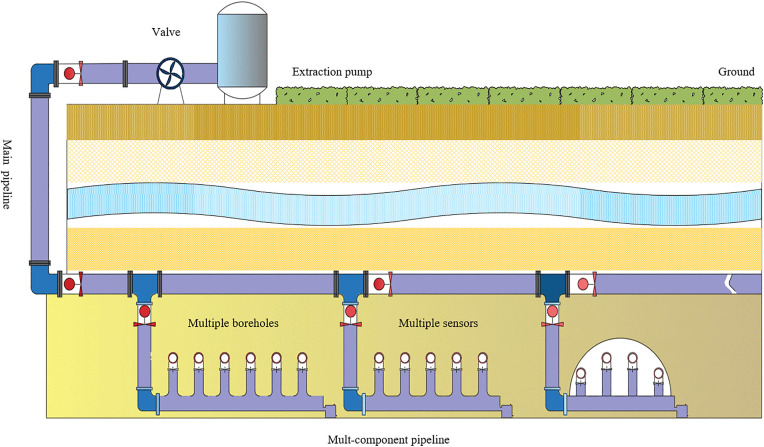
Schematic diagram of a gas extraction system.

A typical gas extraction system comprises a gas extraction pump, a pipeline system, and safety devices. Taking a ground-based fixed gas extraction system as an example, Fig 1 presents a simplified schematic of the entire system.

As shown in Fig 1, the ground-based gas extraction pump serves as the power source for the entire system. Multiple extraction boreholes are grouped into extraction units, and these units form a branch pipeline subsystem. Ultimately, the extracted gas is collected into the main pipeline and transported to the ground. The valve devices installed on each pipeline regulate the drainage process of the entire system. The control tasks of the extraction system are mutually complementary. First, all the preconditions must be satisfied. Second, the gas extraction concentration, the gas extraction purity and gas extraction negative pressure under the optimal state at each moment need to be realized by adjusting the extraction pump power and valve opening. Meanwhile, the pure quantity of gas extraction and power of the extraction pump affect the control target of the gas efficiency ratio. The study addresses this complex task through multi-variable predictive control modelling [29–31].

### 2.2. Optimized mathematical model for gas drainage

Based on theoretical analysis and numerical calculation and under the restriction of safety measures, the optimization mathematical model of high efficiency and energy saving for the gas extraction system is established with the goals of dynamic maximum gas extraction concentration, dynamic maximum gas extraction purity and dynamic optimal extraction pump efficiency ratio, as shown in equations (1)–(3).


$$MAXc(CH_{4}{)}_{t}$$ (1)

$$MAXQc(CH_{4}){}_{t}$$ (2)

$$\eta =\frac{Qc{\left(CH_{4}\right)}_{t}}{W}>\eta_{L}$$ (3)

Under the conditions of safety constraint and efficiency constraint, a mathematical model of gas extraction optimization is proposed to measure the gas extraction effect at every moment so that the gas extraction effect is always maintained within the optimal ideal range. In equations (1), (2), c(*CH*_*4*_)_t_ and Q_c_(*CH*_*4*_)_t_ represent the gas extraction concentration at each moment and the gas extraction pure quantity at each moment, respectively, and the ideal optimal value must be reached at each moment when it is greater than the critical threshold value of the corresponding working condition index. W in equation (10) denotes the current power of the extraction pump. Equation (10) shows that the efficiency ratio of the extraction pump needs to be adjusted at all times to keep it above the minimum efficiency ratio for economic extraction.

## 3. Algorithm design

### 3.1. Intelligent control objectives and tasks of gas extraction

**Fig 2:**
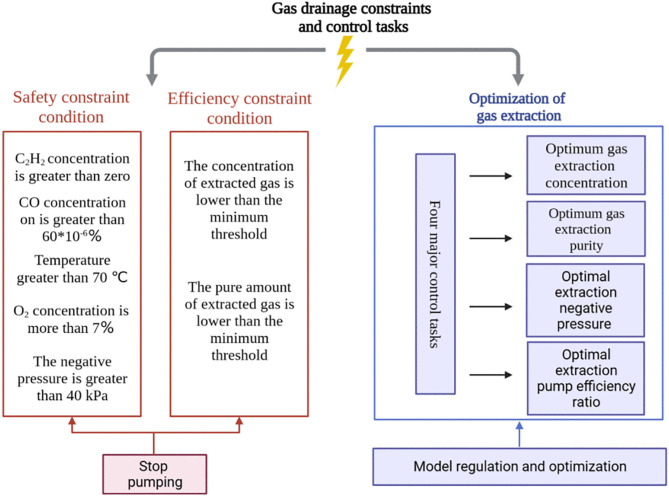
Schematic diagram of the gas drainage constraints and control tasks.

After defining the constraints and optimization objectives, it is necessary to clarify the control objectives under different extraction conditions, which provides theoretical guidance for intelligent control. The control tasks of gas extraction are illustrated in Fig 2.

### 3.2. Prediction model

RNN Based on the gas extraction control strategy, this study proposes an MPC model by adjusting the valve opening and power of the extraction pump to optimize and intelligently control the gas extraction.

(a) Ideal reference curve of the controlled quantity based on simpleRNN

A simple recurrent neural network (simple RNN) is a type of neural network that takes sequence data as input and recurs in the evolution direction of sequence. In this study, the simpleRNN is used to analyze the change information of the historical data of the gas extraction concentration, gas extraction pure quantity, gas extraction pressure and extraction pump efficiency ratio with time, and the fitted change curve is used as the ideal reference curve of model predictive control.

**Fig 3:**
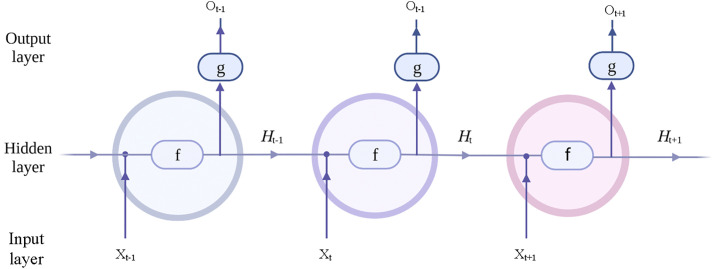
Schematic diagram of the simpleRNN network structure.


Generally, as shown in Fig 3, the simpleRNN network is divided into three layers: input layer, hidden layer and output layer. The data reception of the input layer consists of two parts: hidden layer vector ht-1 at the previous moment and input vector Xt of this layer. Similarly, the data output of the hidden layer has two different flow directions, which are received by the output layer as current output 0t and used as input Ht for the next time. The specific calculation formula at time t is shown in equations (4)–(7).

$$h_{t}=f(U\cdot X_{t}+W\cdot h_{t-1})$$ (4)

$$a_{t}=g(V\cdot h_{t})$$ (5)

$$h_{t}=\tanh(U\cdot X_{t}+W\cdot h_{t-1})$$ (6)

$$a_{t}=soft\max(V\cdot h_{t})$$ (7)

In the above equations, U represents the weight matrix from the input layer to the hidden layer, W represents the weight matrix from the previous hidden layer to the next hidden layer, and V represents the weight matrix from the hidden layer to the output layer. In equations (4) and (5), f and g represent the hidden layer activation function and output layer activation function, respectively. Typically, the tanh function and softmax function are used as shown in equations (6) and (7). The simple RNN propagates errors layer by layer through backpropagation, uses the derivative of the activation function to distribute errors across all units in each layer, and finally updates the weights of each unit based on the correction signals from individual units.

(b) Forecasting model of the control quantity based on MPC

At each sampling moment, MPC solves an open-loop optimal control problem over a finite time horizon to determine the current control action. Due to potential errors between the model’s predicted values and the system’s actual current values—which can reduce the effectiveness of future control outputs—only the first control output in the MPC output sequence is executed.

**Fig 4:**
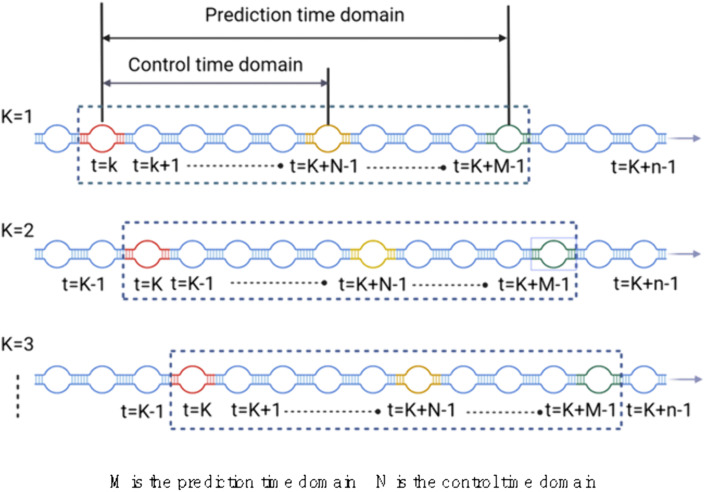
Forecast and control schematic diagram of MPC.

As shown in Fig 4, the horizontal axis represents the time domain. Here, M sampling intervals form a prediction time horizon, and the first N sampling intervals of this horizon form a control time horizon (with M > N). At time t, optimization is performed using the predicted data from the corresponding M sampling intervals, ensuring that the actual values of the controlled variables approach the ideal curves within the N-step control time horizon. The optimal intelligent control strategy for gas drainage in control time domain N is obtained, but only the first decision command of the optimal control sequence in control time domain N is executed. The whole process rolls forward, starting from the initial sampling point K = 1 and ending when regulation time n set by the system is reached, that is, when K = n.

Due to the mutual influence and interaction of various parameters in the gas extraction system, the prediction model based on MPC takes the historical data and future input of the related controlled quantities of the gas extraction system as the model input quantities to further predict the output value of the system control quantities. It adopts a constant coefficient linear discrete state model, as shown in equation (8):


$$X_{t}=AX_{t-1}+BU_{t}+C$$ (8)

In this equation, *X*_*t*_ represents the state vector at the current moment, *X*_*t-1*_ represents the state vector at the previous moment, *U*_*t*_ represents the control input at the current moment, and *C* represents the constant term. *A* and *B* represent the weight matrices of each state vector and each control input, respectively. Assuming that the control input of the future T step is known, the state of the future T step can be obtained, as shown in Equations (9)–(11):


$$X_{t+\text{1}}=AX_{t}+BU_{t+1}+C$$ (9)

$$\begin{array}{c} X_{t+\text{2}}=AX_{t+1}+BU_{t+2}+C\\ \begin{array}{cc} {}*20c & \end{array}=A^{2}X_{t+1}+ABU_{t}+BU_{t+1}+AC+C \end{array}$$ (10)

$$\begin{array}{c} X_{t+T}=A^{T}X_{t}+A^{T-1}BU_{t}+\cdots +A^{T-i}BU_{t+i-1}\\ \begin{array}{ccc} {}*20c & & \end{array}+\cdots +BU_{t+T-1}+A^{T-1}C+\cdots C \end{array}$$ (11)

### 3.3. Correction feedback and rolling optimization

In practical control processes, uncertainties (e.g., nonlinearity, model mismatch, environmental interference) can cause discrepancies between the actual state values and the model’s ideal output values. To enhance the MPC system’s ability to handle uncertainties and improve its anti-interference performance, feedback information is used to implement correction feedback. Specifically, the model prediction error is calculated by comparing the actual measured output values with the model’s ideal predicted values, and this error is then used to correct the model’s predicted values.

MPC is essentially an optimal control algorithm that determines future control effects by optimizing a performance index. Since the control strategy determines the future behaviour, predictive control uses a rolling finite optimization strategy to replace the constant global optimal target, which requires the process output to follow a desired smooth curve to reach a preset reference trajectory. In other words, the optimization process cannot be completed once offline, and the whole process is dynamically and repeatedly performed online. Table 1 shows the RNN and MPC Controller Parameters.

**Table 1:** RNN and MPC controller parameters.

Component	Parameter	Value/Setting
Simple RNN	Input features	Gas concentration (CH₄), seam pressure, pump power, valve opening (4 features)
	Hidden units	128 (single hidden layer)
	Activation function	Tanh
	Sequence length (time steps)	12 (corresponding to 6 hours of 30-min interval data)
	Optimizer	Adam (learning rate: 0.001, batch size: 32)
	Training epochs	200 (early stopping at epoch 142 to avoid overfitting)
MPC	Prediction horizon (*N*_*p*_)	12 (6 hours)
	Control horizon (*N*_*c*_)	6 (3 hours)
	Cost function weights	*Q*(output tracking): diag([10, 5]) (CH₄ concentration, flow rate);*R*(input penalty): diag([1, 2]) (valve opening, pump power)
	Input constraints	Valve opening: 0–100%; Pump power: 200–500 kW (corrected from earlier error)
	Output constraints	CH₄ concentration: < 1.5%; Gas flow rate: > 0.5 m^3^/min

### 3.4. Model construction

**Fig 5:**
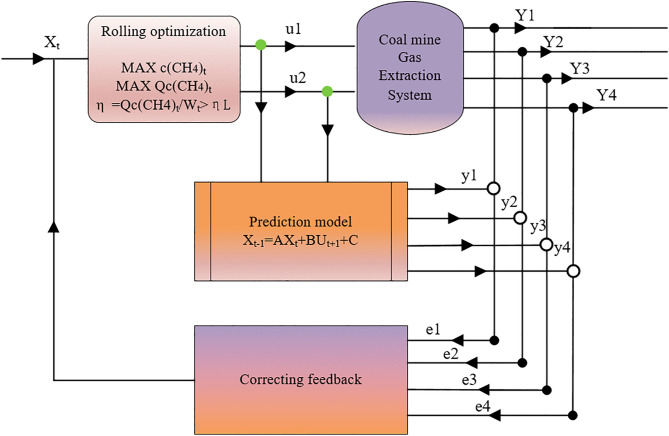
Structural principle block diagram of the predictive controller.

The simpleRNN network is used to study and predict four characteristic variables: gas extraction concentration, gas extraction pure quantity, gas extraction negative pressure and extraction pump efficiency ratio. The fitted curves for future time periods derived from this process serve as the ideal reference curves for the MPC controller. The MPC controller dynamically adjusts the control variables (valve opening and extraction pump power) to ensure that the controlled variables (gas extraction concentration, pure gas extraction volume, gas extraction negative pressure, and extraction pump efficiency ratio) approach the ideal values specified by the reference curves at all times. The structure principle of predictive controller is shown in Fig 5, which includes four main parts: predictive model, rolling optimization, correction feedback and coal mine gas extraction system.

Simulated data was employed as the data foundation for algorithm simulation. The four controlled variables of the gas extraction system are as follows: gas extraction concentration, gas extraction purity, gas extraction negative pressure, and gas extraction pump efficiency ratio. Based on the experience and recommendations of on-site experts, the corresponding control objectives for these controlled variables are defined as follows:

1. Gas extraction concentration: The overall trend of gas extraction concentration shows a decrease from 40% to 5% over time, with the actual value at the current moment being infinitely close to the optimal value of the ideal reference curve.
2. Gas extraction purity: From an overall trend perspective, gas purity gradually decreases over time (with the original range specified as 9.0–5.0 m^3^/min), and the actual value at the current moment is infinitely close to the optimal value of the ideal reference curve.
3. Gas extraction pump efficiency ratio: This ratio should be maintained at approximately 1.5 m^3^/(kW·h) to meet the requirements of economical gas extraction.
4. Gas extraction negative pressure: The negative pressure during the extraction process must be maintained within the safe range of 10–30 kPa.

A set of data samples was collected every 30 minutes, and data changes over a 21-day period were recorded, resulting in a total of 1,000 data points. For clearer and more intuitive presentation, the first 800 data points were visualized. These data include six parameters: gas extraction concentration, gas extraction purity, gas extraction negative pressure, gas extraction pump efficiency ratio, valve opening, and extraction pump power.

The original data underwent cleaning, where missing values and abnormal values were imputed using the nearest mean method. Due to the presence of numerical values with different dimensions in the dataset, normalization was applied to scale the data to the range of 0–1. This step was implemented to enhance prediction accuracy and computational speed. Subsequently, the data was divided into training and testing sets at the conventional ratio of 7:3. A Simple Recurrent Neural Network (SimpleRNN) was utilized to separately process the temporal information in the controlled variable data, thereby obtaining the ideal reference curve.

**Fig 6:**
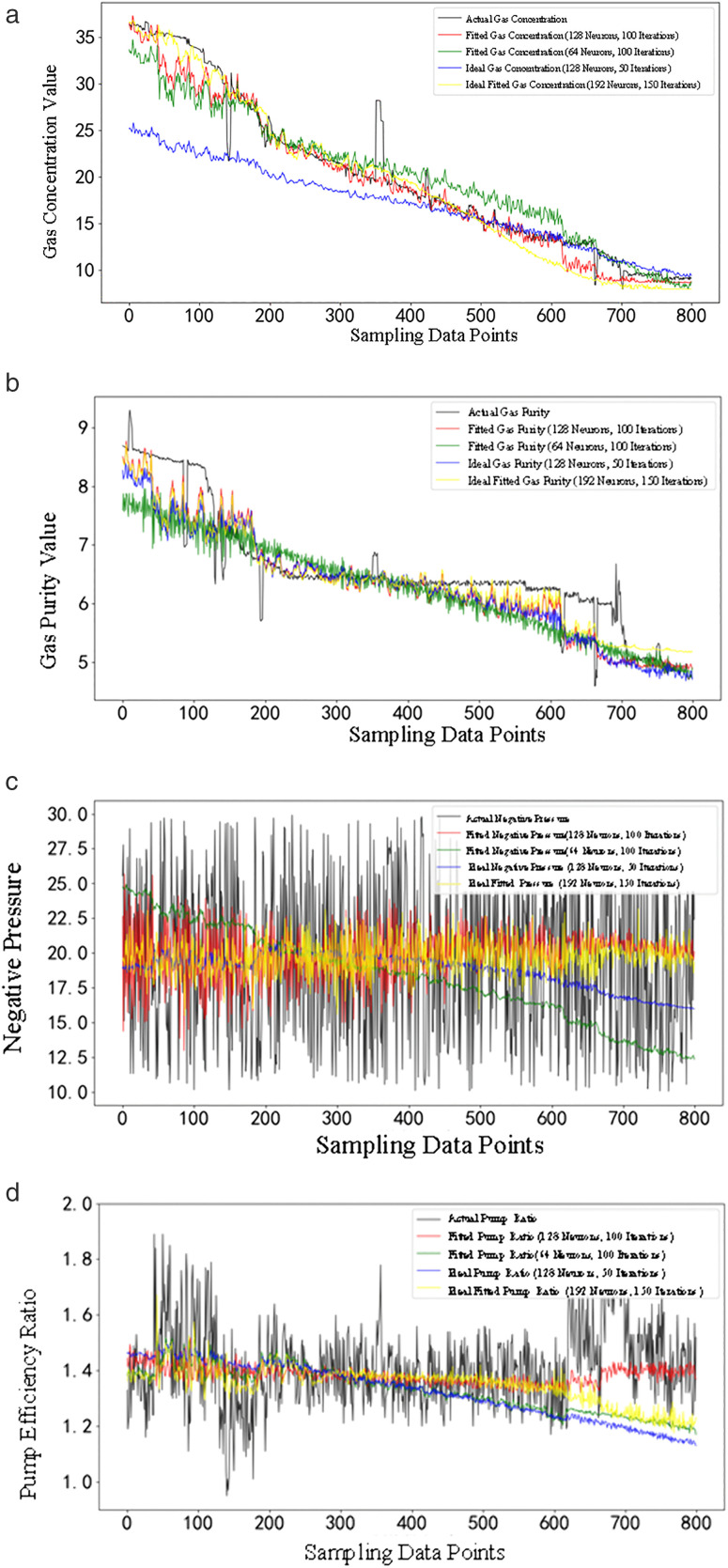
Comparison of Ideal Reference Curve Results for Model Hyperparameter Optimization. **(a)** Comparison of fitting results of gas concentration models; **(b)** Comparison of fitting results of gas purity models; **(c)** Comparison of fitting results of negative pressure models; **(d)** Comparison of fitting results of pumping pump efficiency ratio model.

In Fig 6, subfigures (a), (b), (c), and (d) respectively present the ideal reference curve results for the controlled variables of gas extraction concentration, gas extraction purity, gas extraction negative pressure, and gas extraction pump efficiency ratio. In these subfigures:

The black curve represents the actual (true) data of each controlled variable; The red curve represents the final model-fitted data for each controlled variable when 128 neurons were selected as hyperparameters and 100 iterations were performed; The green curve represents the final model-fitted data for each controlled variable when 64 neurons were selected as hyperparameters and 100 iterations were performed; The blue curve represents the final model-fitted data for each controlled variable when 128 neurons were selected as hyperparameters and 50 iterations were performed; The yellow curve represents the final model-fitted data for each controlled variable when 192 neurons were selected as hyperparameters and 150 iterations were performed.

With regard to the data changes in gas extraction concentration and gas extraction purity, it can be observed from subfigures (a) and (b) of Fig 6 that as the number of hyperparameter neurons and the number of iterations increase, the fitting degree between the model output data and the actual data of each feature improves. Specifically, the red and yellow curves can more effectively represent the variation state of the actual data. However, when the model hyperparameters reach reasonable values, a continuous increase in the number of neurons and iterations does not significantly enhance the fitting degree of the model output data. In other words, compared with the red curve, the yellow curve does not better reflect the variation state of the actual data; instead, it only increases the computational burden of the model and elevates the risk of overfitting.

For the data changes in gas extraction negative pressure and gas extraction pump efficiency ratio, subfigures (c) and (d) of Fig 6 indicate that the red curve not only optimally represents the variation state of the actual data but also maintains the gas extraction negative pressure and gas extraction pump efficiency ratio at approximately 15−25 kPa and 1.3–1.4 m^3^/(kW·h), respectively.

As indicated above, the hyperparameter selection for the model is 128 neurons. Considering both the model complexity and the effectiveness of data fitting, when the number of iterations is set to 100, the model output results are suitable for use as the ideal reference curve for model predictive control, while the model complexity remains relatively low.

## 4. Design of a negative pressure regulation test system for in-seam borehole gas extraction

Physical similarity simulation test are used to study the gas extraction from boreholes in the lower layer, to investigate the changing law of gas parameters (gas concentration, gas pure flow rate, gas leakage) under different extraction negative pressures, to reveal the mechanism of the effect of different negative pressures on the extraction parameters of the boreholes under the influence of gas flow, and to accumulate initial data for the construction of the predictive feedback regulation model and adaptive scheme.

**Fig 7:**
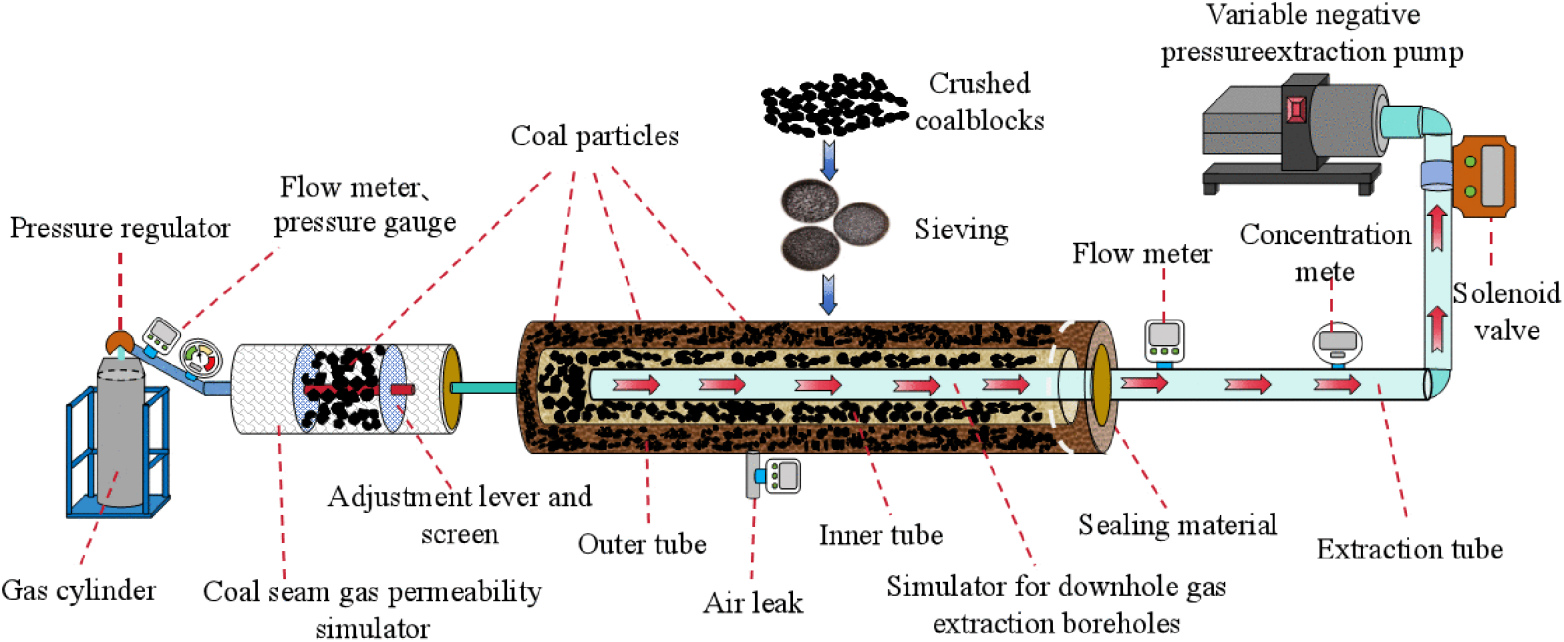
Schematic diagram of the single-borehole negative pressure regulation test system for gas extraction from in-seam boreholes.

This experiment determines the optimal extraction negative pressure to ensure the optimization of borehole gas extraction efficiency by studying the laws of gas extraction parameters changing with extraction negative pressure. In this experiment, a single borehole is taken as the research object to explore the dynamic change laws of gas parameters. To ensure the safety of the experiment, CO₂, which has similar gas flow and adsorption – desorption properties to methane, is used for the research. The test system is shown in Fig 7, which mainly includes gas cylinder, flow meter, coal seam gas permeability simulation device, simulation device of gas extraction borehole in the downstream layer, concentration meter, solenoid valve, variable negative pressure extraction pump and other devices.

The variable negative pressure extraction pump provides the extraction negative pressure. Under the action of this extraction negative pressure, the CO₂ gas in the gas cylinder enters the coal seam gas permeability simulation device and the in-seam gas extraction borehole simulation device to imitate the gas flow within the boreholes.

This device is constructed using a polyvinyl chloride (PVC) pipe with a length of 500 mm and an inner diameter of 50 mm. A steel filter screen is installed at the left end of the pipe, and the middle section is filled with coal particles. An adjustment rod is embedded in the filter screen at the right end to adjust the compactness of the coal particles, thereby simulating different coal seam gas permeabilities. Moving the rod to the right increases the spacing between coal particles, enhancing permeability; moving it to the left reduces spacing, decreasing permeability.

The simulation device for gas extraction boreholes is constructed from coal particles, a gas leakage ring, a simulated in-seam gas extraction borehole, an extraction pipe, and hole-sealing materials. The main part is composed of two PVC pipes of different sizes. The outer tube is 1200 millimeters in length and has an inner diameter of 240 millimeters, while the inner tube is 1100 millimeters long with an inner diameter of 120 millimeters. Device ports are reserved on the outer tube for monitoring the pressure of the coal seam and the air flow. The wall of the inner tube is perforated and covered with a nylon filter screen to prevent broken coal from flowing into the borehole along with the air, thus avoiding damage to the device.

The in-seam gas extraction pipe is a PVC pipe with an inner diameter of 90 mm and a length of 700 mm. Holes with a diameter of 8 mm are distributed on the pipe wall to prevent coal particles from the inner pipe entering the borehole while ensuring unobstructed gas extraction. Coal particles are filled between the three pipes (outer pipe, inner pipe, and extraction pipe) to simulate a gas leakage ring. Air enters the simulated coal seam fractures through gas leakage ports, flows into the borehole under the action of extraction negative pressure, mixes with the CO_2_ flow, and is finally transported to the extraction pump.

An online CO_2_ concentration meter, an MF5702 flow meter, a Hermance joint-type negative pressure meter, and an electric valve are integrated to form a monitoring and control system for gas extraction parameters. The flow meter and CO_2_ concentration meter record changes in gas concentration and flow rate under different extraction negative pressures, while the electric valve enables precise adjustment of the extraction negative pressure.

## 5. Experimental results

Based on the simulation data, the four controlled quantities of the gas extraction system are the gas extraction concentration, gas extraction purity, gas extraction negative pressure and gas extraction pump efficiency ratio. According to the experience and suggestions of field experts, the corresponding control targets of the controlled quantity are the following:

(a) Gas extraction concentration: the overall change trend of gas extraction concentration decreases from 40% to 5% with time, and the actual value at the current moment is infinitely near the optimal value of the ideal reference curve.
(b) Pure gas extraction volume: the overall change trend of gas purity decreases from 9.0 m^3^/min to 5.0 m^3^/min with time, and the actual value at the current moment is infinitely near the optimal value of the ideal reference curve.
(c) The efficiency ratio of the extraction pump is maintained at approximately 1.5 m^3^/(kWh) to meet the requirements of economic extraction.
(d) The extraction negative pressure is maintained in the safe range of 10–30 kPa.

 In the actual process, the adjustable ranges of the control quantity are the following:

(a) The valve opening:0% − 100%.
(b) Extraction pump power:200–500 kWh.

### 5.1. Dynamic fitting of ideal reference curve of controlled variables based on simpleRNN

**Fig 8:**
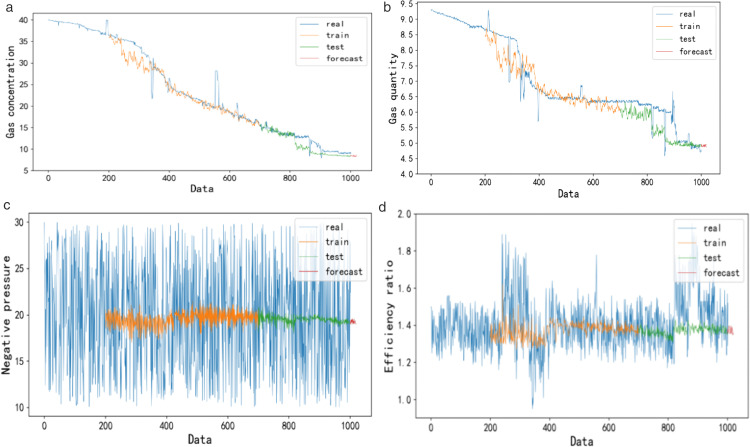
Ideal reference curve of the controlled quantity.

A group of data samples was taken every 30 min, and the data changes in 21 days were counted to obtain 1008 pieces of data. The data included six parameters: gas extraction concentration, gas extraction purity, gas extraction negative pressure, gas extraction efficiency ratio, valve opening and extraction pump power. The original data were cleaned, and missing and abnormal data were supplemented by the adjacent mean method. Because the data contain numerical values with different dimensions, to improve the accuracy of prediction and the operation speed, the data were scaled to between 0 and 1 by a normalization method. Then, the data were divided into the training set and test set according to the conventional ratio of 7:3. SimpleRNN was used to process the time information in the controlled quantity data to obtain an ideal reference curve, as shown in Fig 8.

Fig 8a–8d show the ideal reference curve result diagrams of the controlled quantity gas extraction concentration, gas extraction pure quantity, gas extraction negative pressure and extraction pump efficiency ratio, respectively. The blue part represents the real time series value of the controlled quantity; the orange and green parts represent the ideal output values of the training set and test set, respectively; and the red part represents the ideal output value of the controlled quantity predicted in the subsequent time period after the information extraction of real data. As seen from the figure, the output curve of simpleRNN has a good fitting effect on the true values of the gas extraction concentration and gas extraction pure quantity. The negative pressure of gas extraction and efficiency ratio of the extraction pump are maintained at approximately 15−25 kPa and 1.3–1.4 m^3^/(kW ∙ h), respectively. These curves can be directly used as the ideal reference curves for MPC.

### 5.2. Dynamic adjustment of the control quantity based on model predictive control

**Fig 9:**
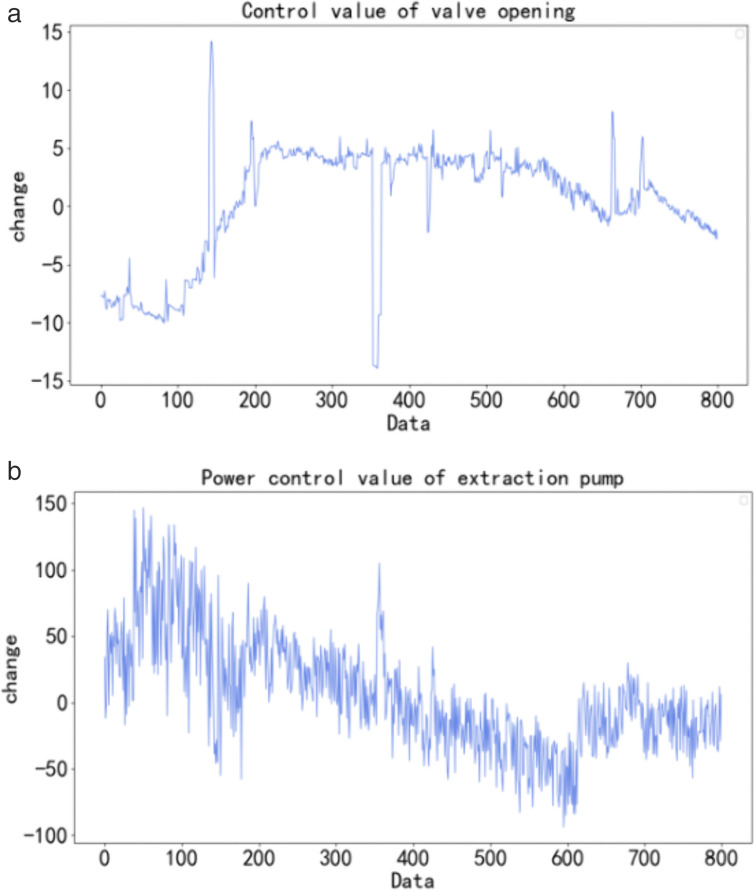
Dynamic control value of the control quantity.

After the ideal reference curve is determined, the control quantity needs to be dynamically adjusted through model predictive control so that the controlled quantity is infinitely near the ideal reference curve at all times. The dynamic control results obtained by using the constant coefficient linear discrete state model are shown in Fig 9.

Fig 9a and 9b show the change in valve opening of the control quantity and power of the extraction pump with time compared to the dynamic control value at the last sampling time. In Fig 9a, the control value of valve opening is between −15° and 15°. In Fig 9b, the control value of the extraction pump power is between 150 kW/h and −100 kW/h. Most of the time, the regulation amplitude is relatively gentle. When there is a large regulation value, Fig 9 collectively show that there is a large difference between the ideal reference curve and the real value at the current time under the interference of environment or nonlinear factors. Thus, the model predictive control approach can effectively regulate and dynamically control the control quantity.

## 6. Conclusion

(a) Given the complex characteristics of gas extraction systems—including multi-variable coupling and strong interference—this study analyzes the prerequisites for gas extraction, including safety and efficiency constraints, and proposes four core control tasks to ensure the safe implementation of gas extraction.
(b) To satisfy these constraints, a simple RNN is used to process the temporal information in the data of four key parameters (gas extraction concentration, pure gas extraction volume, gas extraction negative pressure, and extraction pump efficiency ratio). The resulting ideal reference curves for parameter regulation exhibit excellent data fitting performance.
(c) After obtaining the optimized ideal reference curves for the controlled variables, the MPC algorithm is applied to dynamically adjust the control variables (valve opening and extraction pump power). Through correction feedback and rolling optimization, the control variables are continuously adjusted. Even under the influence of environmental and nonlinear interference, the MPC-based control system achieves superior control effects.
